# Digital biomarkers for Alzheimer’s disease: the mobile/wearable devices opportunity

**DOI:** 10.1038/s41746-019-0084-2

**Published:** 2019-02-21

**Authors:** Lampros C. Kourtis, Oliver B. Regele, Justin M. Wright, Graham B. Jones

**Affiliations:** 1 Clinical & Translational Science Institute, Tufts University Medical Center, 800 Washington St, Boston, MA 02111 USA; 2 Evidation Health, 167 2nd Ave, San Mateo, CA 94401 USA; 3 Cambridge Innovation Center, Eli Lilly and Company, 450 Kendall, Cambridge, MA 02142 USA; 4 Present Address: Massachusetts Institute of Technology, Cambridge, MA USA; 5 Present Address: Novartis Pharmaceuticals, East Hanover, NJ USA

**Keywords:** Prognostic markers, Diagnostic markers

## Abstract

Alzheimer’s Disease (AD) represents a major and rapidly growing burden to the healthcare ecosystem. A growing body of evidence indicates that cognitive, behavioral, sensory, and motor changes may precede clinical manifestations of AD by several years. Existing tests designed to diagnose neurodegenerative diseases, while well-validated, are often less effective in detecting deviations from normal cognitive decline trajectory in the earliest stages of the disease. In the quest for gold standards for AD assessment, there is a growing interest in the identification of readily accessible digital biomarkers, which harness advances in consumer grade mobile and wearable technologies. Topics examined include a review of existing early clinical manifestations of AD and a path to the respective sensor and mobile/wearable device usage to acquire domain-centric data towards objective, high frequency and passive digital phenotyping.

## Introduction

Alzheimer’s Disease (AD) represents a major and rapidly growing burden to the healthcare ecosystem. In the USA alone, some 5 million people suffer from the disease that costs the managed healthcare system in excess of $250 billion. Currently the sixth leading cause of death, AD prevalence has increased by 89% since 2000, underscoring the need for interventive and preventative measures. Despite enormous capital investments, drug development has been problematic. It is generally accepted that the likelihood of reversing anatomic and physiologic changes (e.g., neuronal death) decreases dramatically as the disease advances, placing increased attention on early cohort discovery and patient stratification for any future clinical studies. Accordingly, there is an acute need to detect the disease at prodromal stages. In this quest for monitoring biomarkers for AD assessment, there is growing interest in the identification of readily accessible digital biomarkers, which leverage widely available mobile and wearable technologies, and it is these that are the subject of this review article.

A growing body of evidence indicates that cognitive, sensory and motor changes may precede clinical manifestations of AD by several years.^1^ In particular, sensory and motor (non-cognitive) changes can help detect a neurological or neurodegenerative disease 10 or 15 years prior their effective diagnosis. This said, existing validated neuropsychological/cognitive tests designed to diagnose neurodegenerative diseases are often less effective in detecting deviations from normal cognitive trajectory in the earliest stages of the disease. Furthermore, cognitive tests can suffer from intrinsic cultural bias, take a relatively long time to administer, provide only episodic information, show “practice effect” or “ceiling effect,” and are rater dependent.^2^ Explorations into the inclusion of genetic testing, structural MRI imaging and PET molecular imaging of beta-amyloid and tau protein promise earlier detection of disease, though these tests are currently limited to research applications due to their cost and invasive nature. These limitations preclude repeated and frequent use to test an individual and specifically in the early pre-symptomatic stage.

Mobile and wearable digital consumer technology has the potential to overcome these limitations, and their application in AD detection has become an area of increased interest.

## Mobile and wearable device- derived data

Mobile and wearable technologies (such as smart phones, tablets, smart watches, and rings, smart suits) present a unique opportunity to massively detect neurodegenerative diseases in a timely and economical fashion due to:

a) the widespread usage of such technologies

b) the immediate access of information due to the inherent connectivity

c) the increasing sensitivity and plurality of onboard sensors

d) the nature of these sensors that are uniquely equipped to study such physical and cognitive abilities or symptoms

e) the extremely low burden on the healthcare system, since these devices are increasingly in use by large segments of the population.

Onboard sensors at the heart of these systems are able to provide metrics by means of active (prompted) or passive (unnoticed) measurements, offering considerable flexibility in approach.

*Active data collection* occurs when a user is prompted to perform a measurement and/or enter a metric value, e.g., a digital e-assessment cognitive test that probes memory on a tablet to detect AD,^3^ or a prompted voice test that probes vocal cord tremor to detect PD.^4^ These measurements are usually targeted at addressing specific metrics that have previously been correlated with the disease.

*Passive data collection* occurs when metric values are acquired unbeknownst to the user, e.g, a smartwatch-based step counter that continuously estimates step symmetry and length or a smart ring-based continuous heart rate monitor that picks up heart rate variability (HRV). As such, daily interaction with mobiles/wearables can result in a rich, high-frequency longitudinal data set that can be mined for signatures of a disease—while using users as their own control. Passive data collection has several advantages, including,

- high frequency or even continuous data acquisition,
- objectivity (not influenced by user perspective and learning effects),
- low patient burden, which can lead to higher adherence.

On the other hand, passive data collection may

- be limited to particular metrics that can be collected non-actively,
- be expensive computationally and storage wise,
- requires complex analysis tools to extract useful information.

These issues notwithstanding, given the decline in cognitive function and memory experienced by AD patients, passive data collection provides a logical approach for developing methods for disease forecasting, detection and monitoring. An enormous opportunity is presented for technology developers and healthcare professionals to ideate on new clinical studies which can provide insights to both disease detection and symptom assessment.

**Fig. 1:**
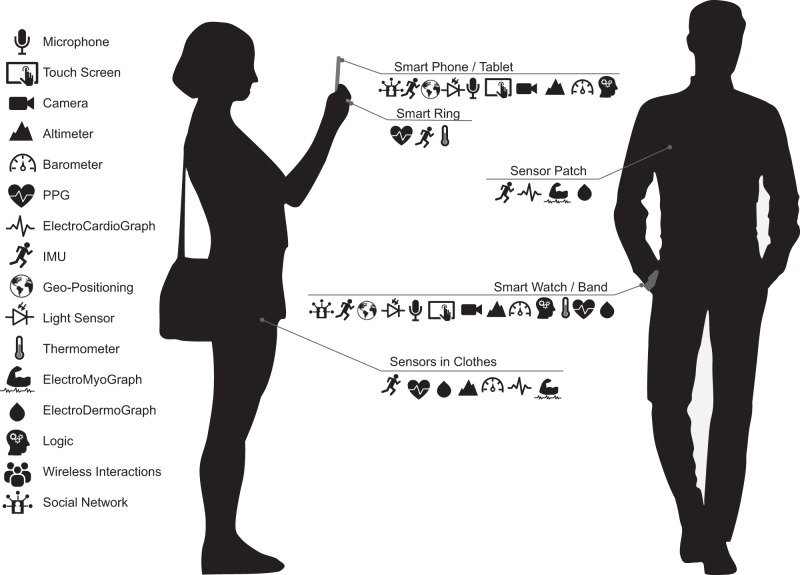
Consumer wearable and mobile devices offer a large personalized, direct, and high frequency sensing potential. Microphones can sense ambient noise and voice. Touch screens can probe for fine motor skills in swiping and typing. Cameras can register eye movements, gaze, and pupillary reflexes as well as capture facial expression traits. Altimeters offer useful information with respect to activity and barometers provide atmospheric pressure readings and weather data. PPG (Photoplethysmography) provides beat-to-beat heart rate measurements (HRM), heart rate variability (HRV) and oxygen saturation (SpO_2_). IMU (Inertia Measurement Unit) includes accelerometer, gyroscope and magnetometer (9 spatial values) and is used by numerous applications to track activity. Geopositioning sensors (GPS and WiFi localization) provide accurate location information. Light sensors read ambient visible or UV radiation levels. Thermometers on rings, patches or watches provide body temperature readings. Electromyograph sensors (EMG) found on patches or suits yield muscle group activity signals. Electrodermograph (EDG) or Galvanic Skin Response (GSR) sensors equip patches and watches to measure the skin conductance and potential or the skin resistance/impendance. Social interactions can be monitored using proximity to Bluetooth or Wi-Fi enabled devices as well as by monitoring overall phone use (calls, texts) and social network activity. Finally, wearable/mobile devices are equipped with logic components that can probe the executive function and memory of a user

This review article considers disease-relevant aspects of sensor and device design, data collection modalities, and a path to clinical grade digital phenotyping. A non-exhaustive summary of available sensors or digital senses on each wearable/mobile device is presented in Fig. 1.

## Disease specific metrics and sensors

*Sensor signals* can individually or collectively provide *metrics* that can determine aspects of a *domain* that is affected by a particular disease or condition (Table 1)

**Table 1:** List of sensors and their respective domains and metrics

Sensor	Metrics	Sense—Domain	Reference
Camera	Saccades, saccades in reading	Occulomotor—eye movements	^5, 30– 32^
	Novelty preference	Occulomotor—eye movements	^22, 27^
	Constriction reflex in response to stimuli	Occulomotor—pupillary response	^12, 23, 29^
	Multiple face features	Behavior—facial expressions	
Microphone	Voice power spectrum and tremor	Speech and language—voice features	
	Vocabulary, syntactic and semantic qualities, pauses, others	Speech and language—cognition	^7, 18– 20^
	Ambient noise level—dominant frequencies	Environment	
Accel/gyro	Gait metrics, distance, steps, symmetry, etc	Movement—gross motor	
	Other activity metrics (bike, swim, run, etc)		^6– 10^
	Overall activity level—energy consumption		^79, 84^
	Tremor	Movement—fine motor	^9, 13– 17^
Barometer	Gait/climb information	Movement—gross motor	
	Barometric pressure	Environment	
Touchscreen	Swipe pattern efficiency	Movement—fine motor	
	Keyboard typing/tapping speed		^9, 13– 17^
	Vocabulary, syntactic, and semantic qualities	Speech and Language—written text	
Geoposition	Location patterns	Behavior and movement—mobility and patial memory	^73, 78^
	Driving patterns and navigational efficiency	Executive function—reaction time and spatial memory	^82, 83^
	Activity level	Movement—actigraphy	^79, 84^
Device use	PIN and password attempts, reminders use, and more	Executive function—memory	^92^
	Number of apps used, frequency, use patterns	Executive function	^85– 89, 93– 95^
	Behavioral disruptions, social circle size, frequency of interactions	Behavior—social interactions	^49, 64– 69, 71, 72, 74– 77^
ECG	Heart rate (HR) and heart rate variability (HRV)	ANS function—heart electric activity	
	System recovery metrics		^36– 39, 99^
	Sleep patterns, phases and efficiency		
	Electrical activity metrics		
PPG	Heart rate (HR) and heart rate variability (HRV)	ANS function—systemic circulation	
	System recovery metrics		^36– 39, 99^
	Sleep patterns, phases and efficiency		
	Oxygen saturation (SpO_2_)		
IR thermometer	Skin temperature	Metabolic activity and hormonal Cycle	
		Immune system health, Acute illness	
Ballistocardiography*	Sleep patterns, sleep phases and efficiency	Sleep	^34, 53– 57, 100^
Galvanic skin response	Skin electrical resistance	Behavior—emotional stress levels	
		ANS function—physical stress levels	
Ambient light sensor	Light intensity at visible wavelength	ANS function—circadian rhythm	
		Environment	
UV sensor	Light intensity at UV wavelength	Environment	
Electromyogram (EMG)	Activity level	Movement—gross and fine motor	
	Tremor	ANS—neuromuscular system health	
	Seizures		

*Ballistocardiography data acquired using a mattress strip, a non wearable

### Movement—gross motor function. Sensors: IMU, geopositioning

A majority of AD patients exhibit pyramidal and extrapyramidal motor impairments starting at an early stage of the disease, which precede signs of cognitive impairment by at least a decade.^5^

*Gait* speed, stride length and gait symmetry are statistically reduced and gait speed variability is increased.^6^ This can be measured actively, by performing a fixed distance or duration walking test, or passively, by monitoring a subject using fixed^7^ or portable equipment.^8^ Buracchio and colleagues^9^ found that there is an inflection point in longitudinal observations of gait speed 12.1 years before clinical diagnosis of MCI (Mild Cognitive Impairment) where the annual decline rate in gait speed goes from −0.005 m/s/y to a dramatic −.023 m/s/y. It has also been shown that central nervous system impairment is related to stance time variability, whereas sensory impairment is related to step length variability.^10^

Although gait metrics alone provide limited specificity for AD detection, their value could come by incorporation into a composite scoring system. IMU sensors on smart phones, watches, rings and patches can estimate such metrics with good accuracy (step count: −6.7 to 6.2% for smartphone apps; Stride length: <5% for median values).^11^ In addition, contact pressure sensors (sock, shoe) can provide even higher insight on gait characteristics such as stance/swing ratio (pressure correlation > 95%).^12^ Further accuracy improvements can be achieved by fusing geopositioning information. Longitudinal monitoring of these metrics can help create composite disease predictors.

### Movement—fine motor control. Sensors: touch screen, keyboard & stylus

*Fine motor* control and more particularly finger tapping speed and tracing accuracy have long been probed as potential early signs of AD. The finger tapping test is an active test where the subject is asked to tap beat a button as fast and as regularly as they can for a period of time; the total number of taps is recorded. While tapping speed normally decreases with age at a rate of −0.03 taps/y, the speed after the inflection point (2.66 y prior to clinical manifestation of the disease) dramatically decreased to a rate of −0.15 taps/y.^9^ Rabinowitz and colleagues^13^ showed that the contact time in a tapping test for subjects having an MMSE < 23 (a cognitive test used to evaluate AD: 20−24 is considered mild dementia, >24 is considered Normal) was increased by 38%, suggesting a much slower reaction speed. Tapping tests have already been implemented in smart phone applications with good patient adherence^14^ with a primary focus on Parkinson’s disease monitoring. Moreover, finger tapping speed has been correlated (*r* = 0.77) to inter-keystroke interval (typing speed),^15^ hence the potential for high frequency data collection from daily computer/tablet keyboard use. More recently, it has been shown^16^ that the text keystrokes per minute (excluding non-text keystrokes) as well as the number of pauses while typing, can discriminate between cognitive impairment (128.48 ± 35.03 keystrokes per minute) and healthy controls (63.65 ± 32.64 keystrokes per minute). Fine motor control can also be probed by looking at the accuracy of a digital pen motion, as in a tracing test, administered with a digital pen and tablet; the standard delineation (RMS distance) from the actual shape is calculated^17^ and was found to correlate with visuomotor performance and age.

Since one of the main means of interaction with mobile equipment is typing or drawing using a stylus, probing for typing speed and pauses while typing as well as pen trajectories present an excellent opportunity to longitudinally evaluate early signs of AD.

### Speech and language. Sensor: microphone

Many aspects of language including grammatical and informational content as well as speech characteristics show deficits with increased AD progression. Using a combination of metrics such as periodic and aperiodic segment lengths, vocal reaction time, relative length (question/response), the amount of insertions/deletions and other irregularity traits, Konig and colleagues^18^ were able to classify between healthy controls, MCI and AD patients with an success rate of up to 87%. Fraser showed that the use of semantic, syntactic and acoustic voice features in a short picture-describing narration test can increase specificity of the disease and its stage.^19^ Even the simple metric of quantifying between-utterance pauses was shown to correlate with episodic memory that is associated to AD.^20^ In another simplified metrics study,^21^ the proportion of spoken words in a discussion (user vs interlocutor) was shown to positively correlate with transitions from normal cognition to MCI in the pre-symptomatic phase.

Conversations over mobile phones or between user and digital assistants are an excellent source of dense speech input. The available automatic speech recognition technologies (for example Google Assistant, Apple Siri, Microsoft Cortana, Amazon Alexa) claim high accuracy and can be used for transcription of subject discussions to be further analyzed. Another good source of language metrics is the keyboard-entered text on a mobile phone or tablet. As such, the syntactic and semantic analysis of spoken or written language can reveal early signs of the disease in a longitudinal, passive manner.

### Occulomotor. Sensors: camera, light sensor

Eye movements and pupillary reflex have been used for several decades in neurological disease research. Careful examination of both allows to probe the medial temporal lobe memory system,^22^ the cholinergic neuronal pathways,^23^ the progressive neuropathological changes within the newcortex^24,25^ and the brain dopamine activity.^26^

#### Visual preference

The Visual Paired Comparison active test is administered by presenting on a screen a series of image pairs to a subject; these pairs include images that have previously been shown to the user.^27^ Healthy control eyes consistently perform more fixations on the novel image, whereas pre-AD subjects do not.^22^ Given the amount of new information we all receive from our tablets, a passive test that measures the fixation time on each new or old graphic presented to a user could be devised in order to quantify the extent of neuronal loss in the medial temporal lobe.

#### Pupillary reflex

Pupillary constriction and dilation in response to light intensity changes is an efficient way to evaluate the central cholinergic dysfunction and consists a balance of forces exerted by the iris sphincter and dilator muscles. In an active test developed previously^23,28^ where a light flashes while the subject eyes are recorded using a high speed camera, it was shown that AD patients had significantly lower pupil constriction velocity and acceleration. Similarly, it was shown that pupillary reflex caused by abrupt changes in the illumination in a room were significantly different between patients with AD and controls.^29^ Today’s phone and tablet cameras have enough resolution to capture pupil diameter at high frame rate, thus providing the potential for a high frequency, pupillary reflex passive data collection.

#### Eye movements in reading

Reading is a complex process that involves optical sensory function, cognitive processing of incoming information and occulomotor functions. Using standard text and a high speed eye tracker, researchers showed that patients with early AD exhibited reduced number of words per fixation, an increase in the total number and duration of fixations and and increase in the number of words skipped.^30,42^ Given that reading is one of the basic functions performed on tablets, an on board high frequency eye movement data collection system while text is presented can provide insight to the stage of the disease. In addition, AD subjects show increased latency decreased eye movement velocity, and also have trouble fixating on a target.^31^ In a recent study, AD patients exhibited longer maximum fixation times compared to controls (2908 vs 1951 ms, respectively) as well as a higher number of large intrusive saccades (2.5 vs 0.7 respectively per test).^32^

Eye Blink Rate has also been examined^26^ to be a potential biomarker of Mild Cognitive Impairment, with MCI participants having a higher blink rate per minute than healthy controls (27.60 ± 15.09 versus 20.24 ± 13.24). Given the time spent in front of a tablet or phone screen, longitudinal blink rate changes can be picked up by the face camera and can be used in the digital biomarker arsenal for early disease detection.

Effective eye tracking is possible real time, given the high resolution of tablet and phone cameras. Numerous programs are currently active in developing video-based eye tracking solutions that can be incorporated in future operating systems of mobile devices, probing continuously for metrics that relate to AD.

### Autonomic nervous system function. Sensors: PPG, ECG, ballistocardiography

A key hallmark of AD is the disruption of the cholinergic system of the brain; the resulting acetylcholine deficiencies are tied to many of the higher order cognitive symptoms such as memory loss and attention deficits^21^ and are related to severity of dementia.^28^ This also results in downstream physical symptoms through the disruption of the Autonomic Nervous System (ANS), whose parasympathetic system is heavily dependent on acetylcholine.^33^ Since both cholinergic^34^ and autonomic brainstem nuclei^35^ are amongst the earliest areas of the brain affected by AD-related tau aggregation, preceding cognitive symptoms by years, ANS disruptions represent a compelling opportunity to identify AD early on.

An important marker of ANS balance is Heart Rate Variability (HRV), a measure of the time intervals between heartbeats, resulting from the dual modulation of the heart by the sympathetic and parasympathetic systems. Due to the bidirectional vagal innervation between the heart and the brain, HRV has also been put forward as an index of cognitive function and stress.^33^ In healthy adults, lower Heart Rate Variability (and thus suppressed parasympathetic activity) was correlated with cognitive function,^36^ attention and working memory,^33^ mental stress^37^ and social cognition.^38^ In AD and MCI populations, where Parasympathetic function is suppressed due to damage to the cholinergic systems, HRV has been found to be lower than healthy controls as well as being negatively correlated to the level of cognitive function.^39^ Given the ties between HRV and cognition, the early disruption of the cholinergic and parasympathetic system as well as its progressive decline in AD, HRV serves as a compelling marker for AD progression.

The advent and increased prevalence of fitness trackers and smart watches equipped with photoplethysmography (PPG) capability set the foundation for widespread passive and continuous monitoring of users’ heart rate and heart rate variability.^40^ Although there are varying levels of validity between different devices when compared with ECG,^41^ studies have shown that in certain conditions (e.g., resting still) and for certain devices, HRV measurements were accurate enough to be used as detection tools.^42,43^ Proof-of-concept studies have used the data from devices with significant consumer uptake such as Apple Watche (Apple Inc, Cupertino, CA) and Android Wear (Google Inc., Mountain View, CA) to detect conditions passively such as atrial fibrillation with moderate amounts of accuracy.^44,45^ Smart rings equipped with PPG, such as the Oura ring (Oura Health Ltd, Oulu, Finland), have shown high reliability in measuring HR and HRV when compared to Electrocardiography (ECG).^46^ Consumer Ballistocardiography devices, in the form of sensing pads placed on the mattress, have also been shown to be capable of passively measuring HRV^47^ and detecting arrhythmias^48^ based on HRV signals. Another way to probe for the sympathetic nervous system condition (mainly arousal periods) is to measure skin resistance that varies with the state of sweat glands using a Galvanic Skin Response (GSR) sensor.^49^ A series of wrist-worn devices are equipped with such sensors, Empatica’s E4 (Empatica, Milan, Italy) and Verily’s Study Watch (Verily, South San Francisco, CA) have a validated track record of determining stress/anxiety during activities. Other emerging heart monitor modalities include the use of wrist or finger derived ECG from a wristband or a smartphone (KardiaBand and KardiaMobile, Alivecor, Mountain View, CA; Apple Watch, Cuppertino, CA), demonstrated to detect atrial fibrillation and tachycardia.^50,51^

### Sleep patterns. Sensors: PPG, microphone, IMU, ballistocardiography

A commonly reported feature of AD has been circadian rhythm disruption in the form of sundowning or sleep fragmentation. Sleep studies of AD populations have confirmed these phenomena with sleep lab research indicating that patients with AD experienced more night-time awakenings, less time in REM sleep and lower sleep efficiency.^52,53^ Furthermore, the level of sleep disruption appeared to track with the level of cognitive deficit.^53,54^ Sleep disruption is corroborated by the biological changes in AD as the disease attacks the basal forebrain structures of the cholinergic^55^ and raphe nuclei of the serotonergic systems^34^ that contribute to sleep. Components of these systems are among the earliest affected brain areas and see changes in the prodromal stages of AD before cognitive decline.^56,57^

Indeed, there appears to be a bidirectional relationship between sleep quality and AD as studies have shown that sleep fragmentation contributes to developing Tau and AB pathologies, increasing the risk of developing AD.^58^ Accordingly, sleep quality could serve as an important indication of the early stages of AD.

App based and most wrist worn sleep monitors with a few exceptions, were shown to offer limited reliability at determining sleep stages when compared to Polysomnography (PSG).^59^ At the forefront, the Pulse-On (PulseOn Oy, Espoo, Finland) wearable device and the Oura Ring, have shown high levels of sleep staging accuracy,^60^ and correlation to PSG evaluations.^61^ Ballistocardiography-based sensing pads can automatically stage sleep comparatively to PSG or ECG.^48,62^ Finally, there are consumer Electroencephalography (EEG) headsets that have similar accuracy to PSG.^63^ Implementation of these passive measures to track abundant amounts of sleep data would allow one to capture the subtle, long term sleep deviations that could be indicative of AD related changes long before more blatant cognitive symptoms manifest.

### Neuropsychiatric behavioral disruptions. Sensors: GPS, IMU, Device Usage Log

Beyond declines in specific cognitive and physiological domains, Alzheimer’s disease has also been associated with wider-range disruptions of behavior. Approximately 90% of Alzheimer’s patients experience at least one neuropsychiatric symptom^64^ with a spectrum of resulting behavioral changes such as mood disruptions, agitation and apathy.^65^ Apathy is one of the most common disruptions, affecting up to 90% of patients^66^ and has been implicated in patients’ lessened ability to carry out activities of daily living as well as a decreased motivation to participate in social activities.^64,66^ Depressive features are also common with up to 25% of patients being diagnosed with major depression and 50% experiencing depressive symptoms.^67,68^ Social withdrawal and dysphoria can precede diagnosis by years^69^ and are commonly seen in MCI populations as an early manifestation of AD.^70^ These depressive symptoms are also implicated in the decreased ability to perform activities of daily living and disruption of patients’ routines.^71^ These changes in patient’s life activities manifest themselves in tangible ways, studies have shown decreases in the size of patients’ social networks and frequency of social contact.^72^ These neuropsychiatric disruptions cause early impairment to more complex activities of daily living and can precede the dementia phase.^64^ Similar decreases in time spent outside of the house^73^ and social network sizes^72,74^ were seen in MCI populations. While there was some dispute as to the nature of the relationship between social activity and cognition; some studies showing social activity at baseline was a predictor or risk factor of progression to dementia,^72^ and that the current level of social activity was correlated to current level of cognitive decline.^75^ Continuously monitoring these complex everyday activities may demonstrate the behavioral disruptions resulting from the earliest underlying neurobiological changes.

#### Depression and anxiety symptomatology

Recently, there has been increased interest in using passive data from smartphones and wearables in psychiatry and metal health applications with depressive symptoms being of key interest.^76,77^ Mobility features such as location variance in terms of time and location extracted from the GPS sensors of smartphones have been shown to correlate with depressive symptom severity as determined by questionnaire.^78^ Actigraphy data from the accelerometers on wrist-worn wearables were also able to passively distinguish between subjects with Major Depressive Disorder while passive actigraphy has also been found to be helpful in establishing an objective measure of apathy in diminished activity levels.^79^ Beyond passive measures of physical activity and location, the smartphone also allows for insight into social activity. Meta-information on text messages and conversation frequency have also been correlated with depression severity as well as providing an objective correlate to the Social Rhythm Metric in Bipolar Disorder.^80^ Specific types of smartphone use, non-social (e.g., news consumption) and social (e.g., social networking) have been correlated to anxiety and depressive symptom severity.^81^ While more research needs to be performed in the Alzheimer’s space, these studies indicate the viability of using passive smartphone use data to provide objective measures of subtle neuropsychiatric behavioral disruptions.

#### Driving behavior

AD patients experience spatial confusion and get lost even when around familiar places, resulting in wandering behaviors. Although these symptoms present themselves at later stages of the disease, subtle changes in commuting or executive function patterns may be detected earlier. For example, driving pattern features such as reduced speed (at least 10mph slower) compared to the rest of the traffic, reduced—less than half—mileage overall and a relative increase in the proportion of mileage driven close to home was shown in subjects with early stage dementia.^82,83^ Other metrics include the repeatability of a particular commuting pattern, route tortuosity and the consistency of routine locations on a particular day of the week. A mathematical descriptor of habitual location patterns was proposed by Eagle and associates, by performing principal component analysis on the geo-location vectors and extracting Eigen Behaviors, a digital biomarker of location frequency and intensity^84^: longitudinal excursions from these patterns may indicate an onset of a preclinical manifestation of AD.

### Executive function. Sensors: phone usage log, touchscreen

Alzheimer’s Disease has long been defined by its characteristic decline in cognitive function and thus been evaluated by neuropsychological measures of the following broad cognitive domains: Memory, Attention, Executive Function, Language and Visuospatial Memory.^85^ Although these impairments have traditionally been considered a late-stage phenomenon, there is increasing evidence that cognitive changes in these domains may occur decades before dementia,^86^ specifically memory and executive function,^87^ and attention.^88^ Traditionally, these neuropsychological evaluations have been performed as test batteries or active tasks, typically involving an administrator.^89^ The logistical burden of administering these tests as well as their susceptibility to practice effects^21^ preclude their widespread use in determining a preclinical individual baseline as well as their use in continuous sampling to detect longitudinal deviations. In response to these shortcomings, there has been recent interest in the concept of digital phenotyping and its applications to mental health and psychiatry.^90^

The application of digital phenotyping to neurodegenerative conditions has already shown promising results. Researchers have been able to detect users with neurodegenerative conditions such as Parkinson’s and Alzheimer’s from web search data.^91^ Research at digital health company Mindstrong (Palo Alto, CA) has shown that continuous data from seven days of passive smartphone interactions can predict performance on traditional assessments of memory, language, dexterity and executive function.^92^ However, digital phenotyping in neurodegenerative conditions is still in its infancy as groups have yet to establish a clear, functional link between these passive activities and the cognitive domains of interest. Nevertheless, there are certain passive digital scenarios and evolving associated metrics that appear to tie back to cognitive areas of interest. For example, task-switching or the ability to shift between multiple goals is a component of executive function.^93^ Human Computer Interaction studies have started examining passive user-specific app re-visitation rates^94^ as well as the time-overhead cost from switching between applications^95^ that can be considered a naturalistic example of task-switching. Vigilance, or the ability to sustain attention on a task, is a measure of overall attention^70^ and studies have been able to correlate level of alertness to temporal rhythms of application usage.^96^ While more work is required to further develop and validate these measures across different domains of interest, as well as to apply them in a longitudinal AD study setting, these examples show the potential of passively interrogating cognitive domains from continuous user data.

### Future work: Alzheimer’s disease forecasting using multiple digital senses

Each device *sensor data* stream (e.g., IMU) can be used to define an overall neurological health *metric* (e.g., gait symmetry) for a particular *domain* (e.g., gross motor control or balance). To date, there exists a significant amount of individual *sensor data*→*metric*→*domain*→*disease* validation coming mainly from well-controlled, lab-based clinical observations, some of which are listed in this review article.

Similar metrics, acquired longitudinally and passively, in-the-wild (meaning not in a controlled lab setting), using consumer-grade wearable devices, could produce data that could lead to domain predictors of AD before the actual clinical manifestation of the disease. The overall predictor signal is weak, since the changes in each domain are slow and difficult to separate from normal decline of ageing. Yet, the promise is to further amplify the signal’s ability to forecast AD by combining multiple metrics in a multivariate scoring and, if possible, a detection system. A multivariate approach was recently used to quantify symptom severity in Parkinson’s Disease patients based on mobile devices signals, resulting in a disease severity scoring system.^97^ Given the long time required for AD symptoms to fully manifest, a scoring or classification system could operate by means of anomaly detection, i.e., between user longitudinal trajectories, or/and by means of supervised training. Both approaches would require longitudinal observational studies involving healthy control, converter (to AD) and confirmed MCI cohorts that allow feature extraction of metrics, to inform—or train—the scoring or classification algorithms. In order to establish ground truth in such studies, validation using existing disease assessment methods such as cognitive tests, genomic phenotyping, and ideally longitudinal imaging (volumetric MRI, amyloid PET imaging, tau PET imaging) would be required.

## Discussion

Given the staggering current and escalating projected costs for providing care to AD patients, consumer grade technologies able to detect and monitor, once diagnosed, AD progression represent an urgent need. When developed to full potential, one can envision digital phenotyping of AD becoming a digitally embedded routine practice, triggering a series of interventional measures.

One of the main questions that emerges with such forecasting systems is what to do when a signal is detected. While the debate on the preferred course of action is still on and it involves among others, regulatory, ethical, legal, data privacy and clinical considerations, some options involve:

-Notifying the user that there is something out of the normal with his/her longitudinal rate of progression of neurological health, so he/she can seek further clinical assessment.

-Providing longitudinal disease-related digital biomarkers to a healthcare practitioner, to allow for objective and continuous clinical evaluation of a user.

In parallel, such technologies can be used to establish objective, personalized baseline reference standards to design innovative clinical trials that assess the effectiveness of onset delaying or disease modifying treatment, once available.

An overwhelming amount of work lies ahead before we can claim forecasting and detection of Alzheimer’s disease especially in the preclinical phase, using consumer grade devices, passive data monitoring and analytics. It will require longitudinal, very large population observational studies, to account for inter and intra subject variability. It will also require new ways of securely managing and processing this vast amount of information. Underscoring the potential for such consumer digital devices to impact healthcare, the FDA recently issued guidelines^98^ to provide a clear path and encourage technology developers in their quest for efficient digital phenotyping.
